# Long-Read MDM4 Sequencing Reveals Aberrant Isoform Landscape in Metastatic Melanomas

**DOI:** 10.3390/ijms25179415

**Published:** 2024-08-30

**Authors:** Nehaal Patrick, Michael Markey

**Affiliations:** Department of Biochemistry and Molecular Biology, Wright State University, 3640 Colonel Glenn Hwy, Dayton, OH 45435, USA; nehaal.patrick@osumc.edu

**Keywords:** melanoma, alternative splicing, isoforms, MDM4, long-read nanopore sequencing

## Abstract

MDM4 is upregulated in the majority of melanoma cases and has been described as a “key therapeutic target in cutaneous melanoma”. Numerous isoforms of MDM4 exist, with few studies examining their specific expression in human tissues. The changes in splicing of MDM4 during human melanomagenesis are critical to p53 activity and represent potential therapeutic targets. Compounding this, studies relying on short reads lose “connectivity” data, so full transcripts are frequently only inferred from the presence of splice junction reads. To address this problem, long-read nanopore sequencing was utilized to read the entire length of transcripts. Here, MDM4 transcripts, both alternative and canonical, are characterized in a pilot cohort of human melanoma specimens. RT-PCR was first used to identify the presence of novel splice junctions in these specimens. RT-qPCR then quantified the expression of major MDM4 isoforms observed during sequencing. The current study both identifies and quantifies MDM4 isoforms present in melanoma tumor samples. In the current study, we observed high expression levels of MDM4-S, MDM4-FL, MDM4-A, and the previously undescribed Ensembl transcript MDM4-209. A novel transcript lacking both exons 6 and 9 is observed and named MDM4-A/S for its resemblance to both MDM4-A and MDM4-S isoforms.

## 1. Introduction

Skin cancer is the most common cancer in the United States of America, with estimates predicting that one in five people will develop skin cancer at some point in their lives [1]. Non-melanoma skin cancers (NMSCs) dominate skin cancer diagnoses, with basal cell carcinoma being the most frequent, followed by squamous cell carcinoma, Merkel cell carcinoma, and other rare NMSC [2]. The American Cancer Society states melanoma makes up only 1% of all skin cancers; however, it is the most lethal form, with increasing incidence throughout the world [3,4]. In clinical diagnosis and early stages of the disease, local wide surgical excision of the primary tumor is the most likely treatment of choice [5]. Rapid sequencing of patient tumors has allowed targeted therapy development for use as a first-line or adjuvant therapy based on melanoma patient clinical diagnoses [6].

**Figure 1:**
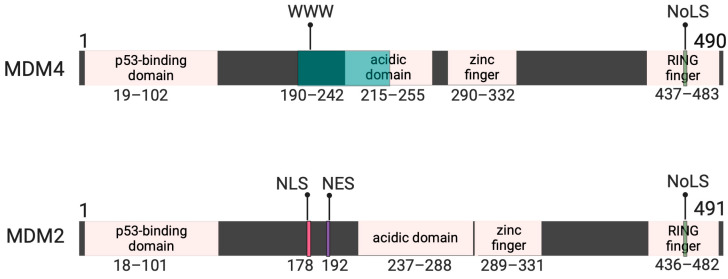
Illustration of the MDM2/MDM4 functional domains in parallel. In MDM4, from N-terminus to C-terminus (left to right), there is a p53 binding domain, the WWW autoinhibitory element that blends into the acidic domain, the zinc finger domain, and the inactive RING finger domain containing the nucleolar location signal (NoLS). In MDM2, from N-terminus to C-terminus (left to right), there is a p53 binding domain, a nuclear localization signal (NLS), a nuclear export signal (NES), the acidic domain, the zinc finger domain, and the active RING finger domain containing the nucleolar location signal (NoLS). Amino acid locations are denoted under each functional domain. Created using previous literature as references for amino acid locations of MDM4/MDM2 functional domains [19,20]. Created with BioRender.com.

The TP53 gene encodes the vital p53 tumor suppressor protein that is frequently referred to as the “guardian of the genome”. TP53 is extremely important in cell cycle regulation; accordingly, it is the most frequently mutated tumor suppressor gene in human cancers. Due to the poor prognosis of tumors harboring TP53 mutations, targeting p53 is an attractive strategy for cancer therapies, such as by restoring the normal transcriptional activity of p53, directly repressing mutant p53 levels, and inhibiting transcriptional targets of mutant p53 [7,8]. Although TP53 is very frequently mutated in human cancers, there is an extremely low p53 mutational rate in melanomas. TP53 is intact and wild-type in over 90% of cases, with less than 10% carrying point mutations; however, it fails to act as a tumor suppressor as it is held inactive in some manner [9,10]. The inactivity of p53 in human melanomas can be attributed to the overexpression of its homologous negative regulators, murine double minute 2 (MDM2, also referred to as HDM2) and murine double minute 4 (MDM4, also referred to as MDMX, HDMX, and HDM4). In fact, p53 is inactive and inhibited by MDM2 and MDM4 in about 50% of all human cancers that retain wild-type p53 [11]. MDM2 and MDM4 share high sequence homology at ~55% and similar functional domains as a result [12] (Figure 1). Data indicate both MDM2 and MDM4 are necessary for proper p53 function and that overexpression of MDM2 and MDM4 contributes to tumorigenesis but in distinct ways from one another [13,14]. MDM2 is thought to mainly regulate the stability of p53 and prevent its accumulation, while MDM4 is hypothesized to contribute more to the regulation of its transcriptional activity [15,16]. Interestingly, MDM4 is overexpressed in ~65% of cutaneous melanomas, whereas MDM2 has lower levels of upregulation in melanomas [17]. The knockdown of MDM2 leads to the overactivity of p53, whereas the overexpression of MDM2 contributes to carcinogenesis by preventing p53 activation, even in times of stress. However, the knockdown of MDM4 may be sufficient and presents a better strategy to restore p53 activity [17,18].

**Figure 2:**
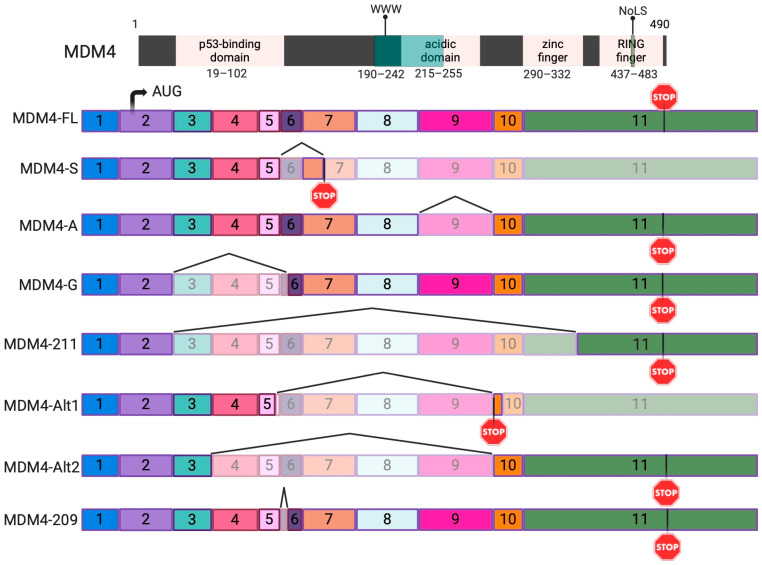
The mRNA of the MDM4 alternative transcripts focused upon in this paper are illustrated here. Exon transparency indicates exon skipping and/or truncated exons. The functional domains are parallel to each isoform and scaled to the missing/retained exons with amino acid locations of each domain. Start codons are represented by AUG, and stop codons are represented by STOP signs. Figure created using Ensembl database and the previous literature as references [25,29]. Created with BioRender.com.

Alternative splicing is a process that allows a diversity of biological responses through the production of different structural and functional proteins diverging from the same pre-mRNA. In mRNA processing, introns are excised by the spliceosome, and exons are linked together in different combinations [21]. Accumulating evidence demonstrates that alternative splicing plays a role in carcinogenesis and drug resistance through the production of tumor-associated isoforms [21]. In melanoma and other cancers, alternative splicing can influence disease onset, progression, and prognosis. One study found that most alternative splicing events were related to patient prognosis in skin cutaneous melanoma and that isoforms play an important role in neoplasia and metastasis using The Cancer Genome Atlas (TCGA) and TCGA SpliceSeq data [22]. The Ensembl database contains 17 transcripts of MDM4, with only 5 having transcript support level one [23,24,25]. The MDM4 isoforms focused upon in this article are represented in Figure 2 alongside the functional domains of MDM4. The two major mRNA isoforms of MDM4 are the MDM4-FL (full length), a stable isoform, and the unstable MDM4-S (short) isoform that excludes exon 6 [26]. The internal deletion of exon 6 (68 base pairs) causes a shift in the reading frame and creates a stop codon within exon 7. MDM4-S mRNA is, therefore, targeted for nonsense-mediated degradation (NMD) [24,27,28]. When exogenously expressed, such as from a plasmid (which does not undergo splicing and, therefore, accumulates no signals for NMD), the MDM4-S protein binds to p53 at a 10-fold higher affinity than the canonical MDM4-FL and is a more potent p53 inhibitor altogether [29,30,31]. The MDM4-A isoform is characterized by the complete removal of exon 9 and 150 base pairs and, therefore, lacks most of the acidic domain, with a partial loss of the autoinhibitory domain. The evidence suggests MDM4-A significantly inhibits p53’s transcriptional activities and is described as oncogenic in melanomas [23,32]. Although the expression of an MDM4-A protein has not yet been demonstrated, previous studies showed that MDM4-A expression positively correlates with poor survival rates in melanoma and is the most commonly expressed isoform in cutaneous melanomas [23].

Traditional NGS short-read sequencing methods produce sequences from ~150 to 300 base pairs (bps) long. NGS has drawbacks in terms of structural variant discovery, sequencing of repetitive elements, and de novo transcriptome/genome assembly [33,34]. Quantification of full-length transcripts using NGS short-read methods is inherently limited due to the lack of sequencing ability and power needed to sequence entire cDNA copies of RNA [35,36]. Therefore, isoform discovery using short reads is limited to the existence of unique splice junctions present within ~150–300 bps of one another within transcripts. This problem is resolved with the use of third-generation sequencing methods that produce long reads spanning entire transcripts. Nanopore sequencing is a long-read sequencing method applicable to whole-genome/transcriptome assembly. This sequencing method can produce reads from 20 bases to 4 million bases long and output up to 35 Gb of data [37]. It allows the unambiguous and accurate characterization of existing splice variants, as well as the discovery of novel isoforms [37].

Therapeutic strategies targeting MDM4 splicing are promising. Several targeting strategies aimed at inhibiting MDM4 expression and MDM4-p53 interaction, as well as inducing the degradation of MDM4 protein, have been proposed [38]. For example, the stapled peptide ALRN-6924 dual MDM2/MDMX inhibitor has shown antitumor activity in phase 1 clinical trials within solid tumors harboring wild-type p53 [39,40]. More specifically, it was discovered that protein arginine methyltransferase 5 (PRMT5) inhibition can reactivate p53 by shifting the alternative splicing of MDM4 toward its unstable MDM4-S counterpart [41,42,43]. PRMT5 was found to specifically downregulate MDM4 protein levels in melanoma cell lines; multiple PRMT5 small molecule inhibitors are currently under evaluation in clinical trials [44]. However, the actual splice variants expressed in melanomas are important to determine, alongside elucidating whether more splice variants exist. Using long-read nanopore sequencing, we identify a novel splice variant of MDM4, as well as the upregulation of an unstudied MDM4-209 isoform. The research methodology is outlined in Supplementary Figure S1.

## 2. Results

### 2.1. MDM4 Alternative Transcripts Expressed in Melanoma Samples

**Figure 3:**
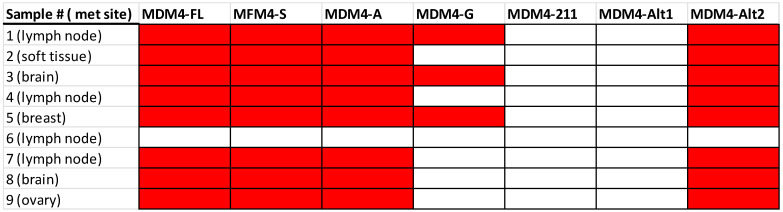
Summary of RT-PCR results in melanoma tumor samples. Red indicates expression of the isoform detected. The tumor site of the melanoma sample is also listed.

For an overview of the MDM4 isoform expression in the melanoma tumor samples, RT-PCR was performed using primers spanning novel exon junctions in each isoform. In Figure 3, the expression and presence of each isoform are indicated. Sample 6 indicated no MDM4 expression and, therefore, serves as a negative control going forward. Apart from sample 6, MDM4-FL, MDM4-S, MDM4-A, and MDM4-Alt2 were expressed in every sample. MDM4-G was expressed in approximately one-third of all melanoma samples. MDM4-211 and MDM4-Alt1 expression were absent in all tumor samples. These data are consistent with the preliminary results, where no MDM4-211 expression was observed in the RT-PCR data of melanoma or nevi specimens, and MDM4-Alt1 expression was detected in only 1/40 specimens [23]. Overall, it serves as a baseline of isoform expression for the expected results in the following sequencing data.

### 2.2. MDM4 Specificity Was Achieved in Overall Sequencing Data

**Figure 4:**
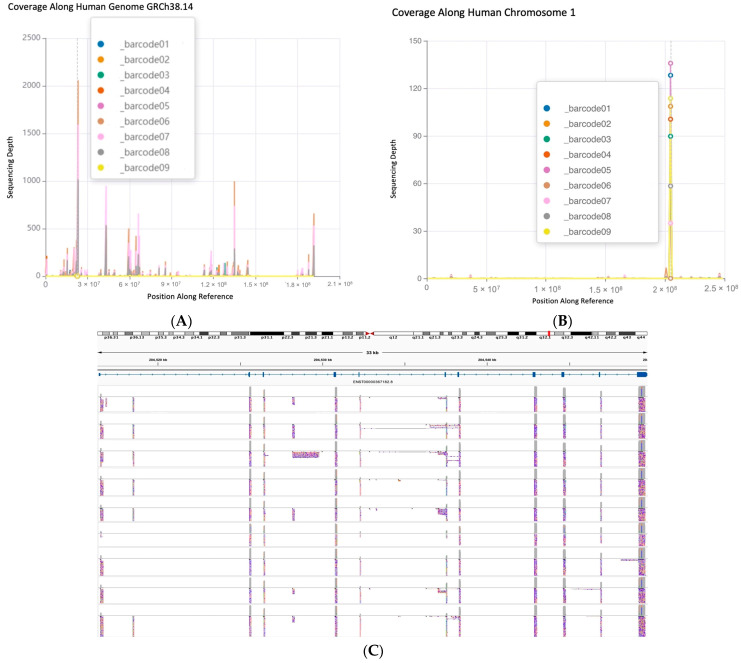
(**A**) Sample alignment coverage along human genome GRCh38.14. All samples were aligned to the FASTA reference of GRCh38.14 from Ensembl database. The *x*-axis corresponds to the nucleotide position in the reference genome, and the y-axis corresponds to the sequencing depth at that locus. Barcodes 1–9 correspond to samples 1–9. The largest spike in sequencing depth for all samples is observed in chromosome 1 around the 2 Mb position, where MDM4 is located within the genomic reference. (**B**) Sample alignment coverage along human chromosome 1. The FASTA reference of GRCh38.14 from Ensembl was trimmed down to a chromosome 1 reference and used for alignment. A single large peak in sequencing depth for all samples is again observed around the 2 Mb position, where MDM4 is located within the genomic reference. (**C**) Visualization of sample alignment depth along human chromosome 1, with representative reads aligned to the exonic sequences of MDM4. The generated BAM and BAM.bai files from the alignment were used for read and coverage track visualization through IGV. The Ensembl MDM4-FL (ENST00000367182) transcript was loaded onto IGV, with its position within chromosome 1 (q32.1) parallel to the sequencing data. The coverage tracks (gray) are loaded on top of representative reads, with sense reads in blue and anti-sense reads in red. Spikes in coverage are observed at exonic sequences of MDM4. The full 9008 bp 3′ UTR in exon 11 of MDM4 is not represented. Figures were created using EPI2ME Labs wf-alignment workflow (Oxford Nanopore Technologies), and images were created using IGV (https://igv.org), version 2.16.0; accessed on 27 March 2024.

Using the Native Barcoding Kit from Oxford Nanopore Technologies (ONT), all samples were multiplexed and sequenced using a MinION R10.4.1 flow cell. The FASTQ passed data output for all samples (samples 1–9 correspond to barcodes 1–9) were processed using the wf-alignment workflow by EPI2ME Labs, with the current human genomic reference (GRCh38.14) from the Ensembl genome browser. A total of 2,047,671 reads were analyzed, with a mean read length of 1189 bps, and 8.8% of reads aligned to the reference, with an 82.2% alignment accuracy. Sample coverage along the human genome can be observed in Figure 4A, with the position along the reference on the *y*-axis and corresponding sequencing depth along the *x*-axis. Sequencing depth refers to the number of times a particular nucleotide or sequence is read, whereas increased sequencing depth indicates confidence in the basecalling accuracy at that position. Coverage refers to the proportion of the reference that has been sequenced at least once [45]. The largest spike in sequencing depth along the position in the reference is observed where MDM4 is located, from ~204.51 mega base pairs (Mbs). The low alignment percentage can be attributed to the extremely large reference file used: GRCh38.14 contains~3.1 giga base pairs (Gb) of data, requires > 11.2 GB of volatile memory, and at least 8.8 GB of processing power to map the nanopore reads [46]. Alignment processes, such as Minimap2, become more computationally challenging as the genome sizes increase [47]. 

However, splitting genome indexes into smaller portions has been shown to increase alignment accuracy and reduce system memory requirements in both short- and long-read data [46,47,48]. Therefore, the samples were then aligned to a chromosome 1 reference, as seen in Figure 4B. The percentage of reads aligned significantly increased with 52.1% of reads aligned with a 93% alignment accuracy. Trimming the reference genome down allows for more accurate mapping quality (MAPQ) scores and alignment accuracy since 50% of the human genome consists of repetitive sequences, with about 89% of these repeats located within introns, allowing for reads to map equally to multiple locations [49,50]. MAPQ scores indicate the quality of the individual read alignment and the probability that a read is misaligned [51]. A large, single spike in coverage is observed in Figure 4B for all samples around the location of MDM4 in human chromosome 1. 

The chromosome 1 alignments were then visually analyzed, using Integrative Genomics Viewer (IGV) to ensure the spike in coverage truly corresponded to MDM4. A compilation of representative images from IGV, with the position along the reference and the reads represented as tracks along the corresponding exonic sequences of MDM4, can be observed in Figure 4C. A coverage track of sequencing depth highlights the exonic sequences of MDM4, with gaps between clusters of reads representing intronic sequences. The Ensembl database MDM4-FL (ENST00000367182.8) transcript was loaded into IGV and shown next to the alignment results, showcasing the position in chromosome 1 that MDM4 spans. Sense reads in blue and anti-sense reads in red are both present as the Native Barcoding Kit from ONT allowed the sequencing of both strands of the amplicons. Tracks along the intronic sequence positions of MDM4 are also present; however, the absent coverage track indicates these reads are of low quality, with low MAPQ scores, and do not significantly contribute to sequencing depth after the default filters. The alignment of reads to MDM4 mRNA indicates that our amplicon library preparation was specific, with the exception of sample 6 (which appears to express no MDM4 by RT-PCR, Figure 3).

### 2.3. Sample Alignment to MDM4-FL Reveals Single Nucleotide Variants and Exon Skipping Consistent across All Samples

Next, a modified MDM4-FL reference of the expected amplicon sequence, based on the primer design, was created and used with samples 1–9 in the same alignment pipeline. Table 1 contains the corresponding total reads, mean read lengths, percentage of reads aligned to the reference, and alignment accuracy percentages for each sample. The overall averages of these statistics are also reported in the table. Sample 9 had over 90% of reads aligned with the overall highest percentage of reads aligned. Sample 6 had the most overall reads but held the lowest mean read length and lowest number of reads aligned. However, this result is consistent with the previous RT-PCR data in which no MDM4 expression was detected. Although only 55 reads aligned, the alignment accuracy by which the reads aligned to MDM4-FL was still congruous with other samples. Sample 6 was included in all analyses. 

**Figure 5:**
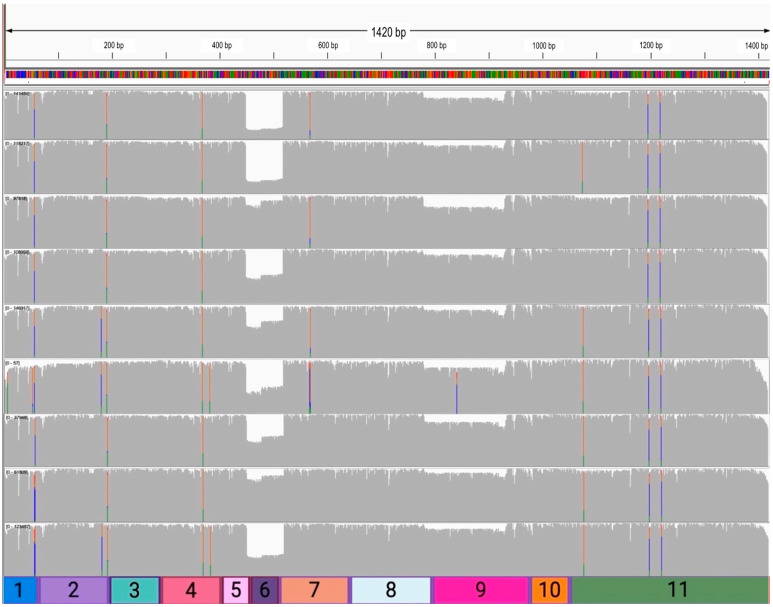
Coverage tracks of sample alignment to the amplicon-specific MDM4-FL cDNA, with corresponding parallel exons. Representative images of coverage tracks from samples 1–9 (top to bottom) using BAM, BAM.bai alignment files, and the trimmed MDM4-FL FASTA reference file used in the EPI2ME Labs wf-alignment workflow (Oxford Nanopore Technologies). The total number of bases in the reference (1420 bp) is located above the coverage tracks, and the corresponding sequence is a colorful bar above the coverage tracks, where each color indicates a unique base: green = adenosine, blue = cytosine, red = thymine, and orange = guanine. The colorful bars within the tracks indicate significant nucleotide variations from the reference sequence within ≥20% of quality weighted reads. Small, bracketed numbers at the top left-hand side of each coverage track represent the number of reads used to create the track. Exons are parallel under the coverage tracks for a rough visual estimate of the position in the reference. Images were created using IGV (https://igv.org), version 2.16.0; accessed on 25 March 2024.

The alignments per sample were then visually analyzed in IGV. Figure 5 contains representative coverage track images of the read distribution from each sample alignment, with corresponding exons in parallel. When nucleotides diverge from the reference sequence in more than 20% of quality weighted reads, colorful bars highlight the mismatches in proportion to the read counts of nucleotides [52,53]. The NCBI dbSNP database of genetic variation has characterized over 18,000 nucleotide variants for MDM4, and SNPs are reported as Reference SNP clusters (refSNPs) with assigned identification numbers beginning with “rs” if multiple SNP entries are input for a single position in the genomic assembly [53]. 

Consistent single nucleotide variants are present within every sample at the ~50 bp (exon 1), ~200 bp (exon 2), ~375 bp (exon 4), ~1200 bp (exon 11), and ~1225 bp (exon 11) region. The exon 1 (chromosome 1, position 204,516,493) SNV (C>T) is characterized as the rs1658982433 variant in the dbSNP database. At the end of exon 2/ beginning of exon 3 (chromosome 1, position 204,526,364), the SNV (G>A/G>C/G>T) is named rs771203637 in the refSNP database. The SNV (G>A) at exon 4 (chromosome 1, position 204,530,790) is named the rs1242872664 variant. Two SNVs are consistent across all samples located within exon 11. The first, at position 204,549,296 within the genome, is a cytosine deletion (rs2102455916) designated clinically relevant in ClinVar with “uncertain significance”. This characterized SNV causes a frameshift mutation; however, it has only been identified once in ClinVar. A non-clinically relevant SNV (C>A/C>G/C>T) is also located at the same chromosome 1, 204,549,296 position, identified as the rs774519008 variant. The second SNV (C>G/C>T) located within exon 11 (chromosome 1, position 204,549,319) is characterized as the rs753233164 variant. 

The minor exon-skipping events were then examined. The individual read “deletions” observed were verified through comparison to the MDM4-FL sequence used in alignment. The major dips in coverage, prominent for all samples, around the 450–525 bps region of the reference sequence perfectly correspond to the loss of exon 6, indicating a surplus of MDM4-S reads. The dips in coverage around the 775–950 bps region of the reference sequence correspond to the loss of exon 9, congruous to the MDM4-A variant. A prominent dip in coverage in the first half of exon 6, compared to the second half, is also present, mainly in samples 3–8. This dip in coverage specifically corresponds to the loss of the first 29 bases of exon 6 and is identified in Ensembl as MDM4-209. MDM4-209 is a non-protein coding transcript designated as TSL 5, indicating it is a poorly supported transcript model in which no single transcript supports the structure of the model [25]. This isoform of MDM4 is undescribed in the literature to date.

### 2.4. MDM4-S Is Overall More Highly Expressed than Canonical MDM4-FL and MDM4-A Isoform in the Tumor Samples

**Figure 6:**
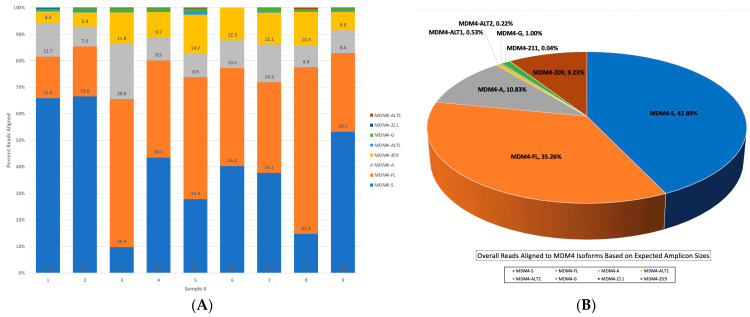
(**A**) The MDM4 isoform identity per melanoma sample. A stacked bar graph representing the percentage of reads aligned to each isoform per sample. The *x*-axis contains the sample numbers, and the y-axis represents the percentage of reads aligned, normalized to 100%. The graph was created in Excel using alignment statistics and reads aligned to each isoform averaged to the total reads mapped per sample, obtained from the wf-alignment pipeline using a custom MDM4 transcriptome FASTA reference (Oxford Nanopore Technologies). (**B**) Overall sample alignment to the amplicon-specific MDM4 transcriptome. A 3-D pie chart representing overall isoform identity of sample alignment results from the wf-alignment pipeline using a custom MDM4 transcriptome FASTA reference. Chart was created in Excel using alignment statistics of overall total reads aligned to each isoform, averaged to the overall total reads mapped for creation of percentage of total reads aligned to each isoform (Oxford Nanopore Technologies).

To determine the isoform identity of each sample, a custom MDM4 transcriptome, based on the expected amplicon sizes of each isoform depicted in Figure 2, was used in samples 1–9 in the same alignment pipeline. The alignment statistics obtained were used to create Figure 6A, containing the individual alignment results of each sample. 

The MDM4-S isoform was clearly overrepresented in samples 1, 2, and 9, whereas the canonical MDM4-FL transcript was the most abundant in samples 3, 5, and 8. Surprisingly, the MDM4-209 isoform observed in coverage track data was expressed in all samples. An even more interesting finding was that the MDM4-209 transcript was almost as equally represented in the alignment results as the MDM4-A transcript. Preliminary studies from this laboratory determined MDM4-A to be the most commonly expressed alternative transcript of MDM4 in melanomas through TCGA data, the Patient-Derived Model Database (PDMDB), and RT-PCR of melanoma and nevi samples [23]. Due to these data, MDM4-A was, therefore, expected to be more highly represented than other isoforms of MDM4, including the canonical MDM4-FL. The MDM4-G, MDM4-211, MDM4-Alt1, and MDM4-Alt2 transcripts are present as a very low percentage of reads, without a significant abundance in any individual sample. 

The overall alignment results to the custom MDM4 transcriptome are represented in Figure 6B. Out of 2,047,671 total reads, 41.7% of the reads aligned to the MDM4 transcriptome, with an alignment accuracy of 91% and an overall mean read length of 962 bps. The low-read alignment percentage can be partially attributed to sample 6, as it was included in all alignment analyses and contained both the highest number of reads overall with the lowest number of reads aligned to MDM4. MDM4-S was the overall most highly expressed transcript in the melanoma samples. Combining these data with previous observations suggests that MDM4-A, while frequently qualitatively present, may not be quantitatively the most abundant. MDM4-A was the third most abundant transcript in samples, followed very closely by the MDM4-209 isoform. The MDM4-G transcript was present in very low levels but more abundant than the MDM4-211, MDM4-Alt1, and MDM4-Alt2 isoforms. 

### 2.5. Quantification of MDM4 Isoforms in Melanoma Samples

**Figure 7:**
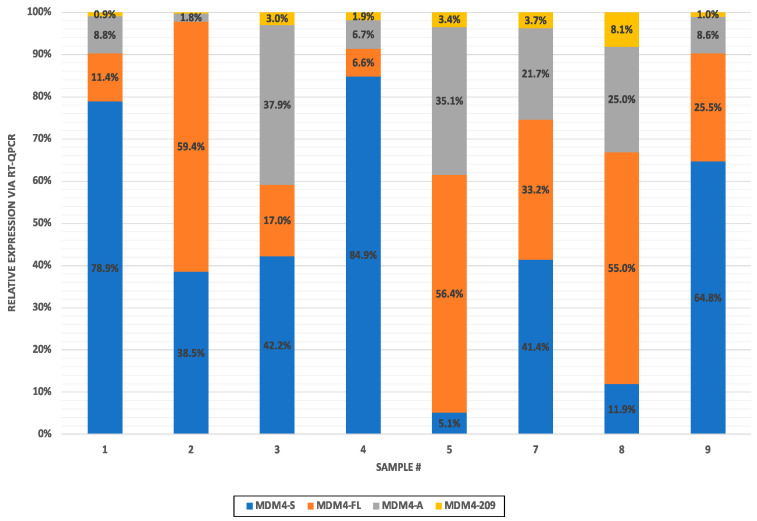
MDM4 isoform quantification via RT-qPCR. A stacked bar graph displaying the relative expression of MDM4-FL, MDM4-A, MDM4-S, and MDM4-209 isoforms presented as 2 ^(−ΔCT)^ and normalized to the overall isoform expression in the sample. The *x*-axis contains the sample numbers, and the y-axis represents the relative expression of the isoform.

Library preparation included the generation of PCR amplicons to increase specificity for MDM4 transcripts. In order to eliminate any possible PCR bias, real-time RT-PCR (RT-qPCR) was performed for all the isoforms represented in Figure 2. A quantitative overview of the MDM4 isoform expression of only the MDM4-FL, MDM4-S, MDM4-A, and MDM4-209 transcripts is shown in Figure 7. All other isoforms were undetectable by qPCR. Sample 6 was omitted due to no isoform detection. 

MDM4-S was more highly expressed than MDM4-A in samples 1, 2, 3, 4, 7, and 9. The MDM4-S isoform was also more highly expressed than the canonical MDM4-FL in samples 1, 3, 4, 7, and 9. In contrast to the sequencing data, MDM4-A was more highly expressed throughout samples in the RT-qPCR data. However, MDM4-209 was expressed at lower levels in the qPCR data compared to the sequencing data. Overall, the amplification bias in the sequencing library was low, as these data are largely consistent; MDM4 isoforms sequenced at low depth were still detected at negligible levels by RT-qPCR. 

### 2.6. Concurrent Deletion of Exon 6 and 9 Reveals Novel Hybrid MDM4-A and MDM4-S Isoform MDM4-A/S

**Figure 8:**
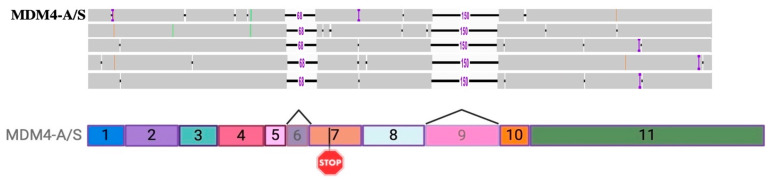
Representation of the novel MDM4-A/S transcript observed in alignment results. The hybrid MDM4-A and MDM4-S transcript with concurrent exon 6 and exon 9 deletions. Representative reads from sample 1 are roughly parallel to an illustration of the isoform. A 68 bp deletion corresponds to the exon 6 deletion, and the 150 bp deletion corresponds to the exon 9 deletion. Small insertions of 2 bps are presented as blue ‘I’s within the read. Image of reads was created using IGV (https://igv.org), version 2.16.0; accessed on 27 March 2024. Exon transparency in the illustration below the reads indicates exon skipping, and stop codon is represented by STOP sign. Created with BioRender.com.

A deeper investigation of read alignment with the amplicon-specific MDM4-FL reference was performed for the visualization of individual reads. A schematic representation of simultaneous exon 6 and exon 9 deletions within a novel MDM4 isoform can be seen in Figure 8, referred to as the MDM4-A/S transcript going forward. An MDM4 isoform with simultaneous deletion of both exons 6 and 9 has not been described in the literature to date. However, reads with concurrent deletions of exons 6 and 9 were present within every sample.

### 2.7. Alignment with Novel MDM4-A/S Transcript Alters Isoform Landscape in Tumors

**Figure 9:**
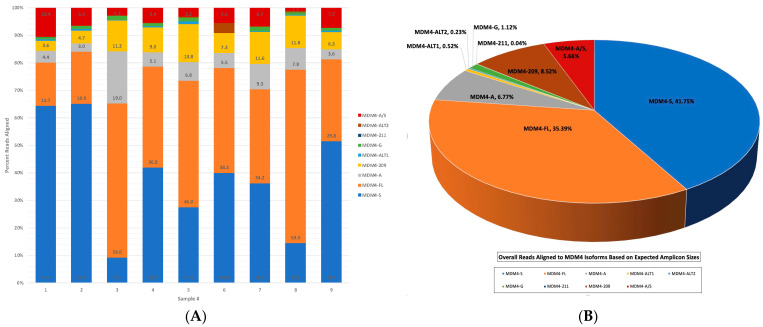
(**A**) The MDM4 isoform identity per melanoma sample, including the novel MDM4-A/S transcript. A stacked bar graph representing the percentage of reads aligned to each isoform per sample. The x-axis contains the sample numbers, and the y-axis represents the percentage of reads aligned, normalized to 100%. The graph was created in Excel using alignment statistics and reads aligned to each isoform averaged to the total reads mapped per sample, obtained from the wf-alignment pipeline using a custom MDM4 transcriptome FASTA reference with a novel MDM4-A/S sequence (Oxford Nanopore Technologies). (**B**) Overall sample alignment to the amplicon-specific MDM4 transcriptome, including the novel MDM4-A/S transcript. A 3-D pie chart representing the overall isoform identity of sample alignment results from the wf-alignment pipeline using a custom MDM4 transcriptome FASTA reference with a novel MDMD-A/S sequence. Chart was created in Excel using alignment statistics of overall total reads aligned to each isoform, averaged to the overall total reads mapped for creation of percentage of total reads aligned to each isoform (Oxford Nanopore Technologies).

Lastly, an MDM4-A/S reference file was created by deleting the exon 6 and 9 sequences from the previous reference and used with samples 1–9 in the alignment pipeline. The new alignment statistics (Figure 9) generally alter the percentages of reads aligned to MDM4-A and MDM4-S to accommodate the new MDM4-A/S isoform. The addition of the MDM4-A/S reference most significantly affected the amount of reads per sample aligning to the MDM4-A isoform. For example, in sample 1, the abundance of MDM4-A reads dropped by 8.3%, MDM4-S reads dropped by 1.6%, and MDM4-209 reads dropped by 0.7%. As the total percentage of reads aligned to the transcriptome stayed the same (41.7%), these reads were allocated to the MDM4-A/S transcript. This led to a higher abundance of the MDM4-A/S transcript at 10.1% over the MDM4-209 and MDM4-A isoform in sample 1. Overall, the MDM4-A/S isoform made up 48,396 reads or 5.7% of the total sequencing data. The overall abundance of the MDM4-A transcript dropped from 11% to 6.7%, MDM4-S dropped by 1%, and MDM4-209 dropped by 0.5%. This novel MDM4-A/S transcript was also found to be more abundant than the isoforms previously described and studied in the literature, including the MDM4-G, MDM4-Alt1, MDM4-Alt2, and MDM4-211 isoforms. 

## 3. Discussion

Melanoma risk factors range from epidemiological factors to external risk factors, such as UV ray exposure, and internal risk factors, such as genetic predisposition [3]. The TP53 gene is the most frequently mutated tumor suppressor gene in human cancers; however, it is intact and wild-type in over 90% of melanomas [9,10]. This inactivity can be attributed, in part, to the frequent overexpression of MDM4 in melanomas. MDM4 is known to enhance MDM2 ubiquitination activity toward p53 and correlate with poor patient survival, and its relatively unexplored p53-independent oncogenic functions make it an important target for research and the discovery of therapeutic targeting strategies [17,23,54,55]. Alongside this, the alternative splicing of MDM4 also plays a key role in melanomas due to the possible oncogenic roles of its isoforms. MDM4-A has been previously discovered to be oncogenic, further highlighting the importance and consideration of MDM4 isoform expression in melanomas and inhibitor development [23]. However, other variants may be favorable for the inhibition of melanoma proliferation. The MDM4-S isoform undergoes NMD and may lower total MDM4-FL protein levels to re-activate p53. The re-activation of p53 by MDM4 inhibition is promising; however, the characterization of MDM4 splice variants expressed in melanomas is also important. More specifically, the consideration of these isoforms in therapeutic target development as alternative splicing events are important factors within cancer oncogenesis. The identification of novel isoforms using high throughput methods, such as nanopore sequencing, is necessary for cancer therapeutic development, as splicing changes can be utilized as biomarkers in clinical malignancy monitoring [56]. Importantly, MDM4 overexpression has been observed in several malignancies with a correlation to pathological prognostic staging in patients [57]. A recent study also characterized the association of MDM4 isoforms with tumor progression and outcome [57]. The current study utilized RT-PCR, RT-qPCR, and long-read nanopore sequencing for the investigation of MDM4 isoforms expressed in malignant melanoma tissue samples. 

Herein, RT-PCR established isoforms expressed in each sample for a baseline of isoform presence in the tumor samples. With the exclusion of sample 6, which was determined to have extremely low levels of MDM4 expression, all samples were expected to manifest MDM4-FL, MDM4-S, MDM4-A, and MDM4-Alt2 and one-third to express MDM4-G. Performing RT-qPCR allowed a quantitative view of isoform expression where, surprisingly, MDM4-S was highly expressed in many samples. It was present at higher levels than the MDM4-A isoform in all cases except 5 and 8, in which high levels of MDM4-FL were present, which has been previously characterized as the most abundant MDM4 isoform in melanomas [23]. This finding does, however, correlate with a previous study that named MDM4-S overexpression a consequence of tumorigenesis rather than a cause of tumor evolution [58]. MDM4-S is also observed as a possible therapeutic target, where switching splicing to increase the MDM4-S/ MDM4-FL ratio may decrease the total MDM4-FL protein for the re-activation of p53 apoptotic activity [23,28]. The elusive function of this isoform alone further highlights the importance of MDM4 alternative splicing research in cancers that retain a wild-type TP53 gene. 

Through long-read nanopore sequencing, MDM4 specificity was confirmed. Genomic alignments detected some off-target reads. Reads aligning to the intronic sequences of MDM4 were present but not in quantities high enough to demonstrate much sequencing depth in the coverage track. Through alignment workflows using an MDM4-FL reference, common existing SNVs and major exon-skipping events corresponding to the loss of exons 6 and 9 were observed as physical dips within the coverage tracks. Interestingly, several reads contained the simultaneous loss of exons 6 (MDM4-S) and 9 (MDM4-A), suggesting the existence of a novel hybrid MDM4-A/S transcript. Since the deletion is approximately 261 bps apart, typical NGS methods relying on short-read isoform discovery through the presence of unique exon junctions within ~150–200 bps of one another failed to identify this transcript. This novel MDM4-A/S isoform is expected to behave similarly to the MDM4-S isoform. Since MDM4-S is hypothesized to undergo NMD through the loss of exon 6 and the subsequent creation of a stop codon at exon 7, the same phenomenon is hypothesized to occur in this novel transcript. An additional surprising finding was the dip in coverage corresponding to the loss of the first half of exon 6, which indicated significant expression of the MDM4-209 non-protein coding and unresearched isoform. MDM4-209 expression was also observed by RT-qPCR and quantifiable throughout all samples. This MDM4-209 transcript is likely to be a target of nonsense-mediated decay; the truncation of exon 6 shifts the reading frame and creates a new stop codon in exon 7. Through alignment to a custom MDM4 transcriptome, overall, MDM4-S was the most highly expressed isoform in our samples. MDM4-A and MDM4-209 also had similar levels of expression. The sequencing results correlate to the RT-qPCR results where MDM4-S and MDM4-FL were both highly expressed throughout the samples. RT-qPCR was performed to eliminate possible amplification bias in the sequencing data. Very high levels of MDM4-S compared to MDM4-FL were still observed. In the RT-qPCR results, however, MDM4-A was present at higher levels compared to the sequencing results. The higher levels of MDM4-A expression in qPCR data may be attributed to the existence of the novel MDM4-A/S transcript. Following alignment to a custom MDM4 transcriptome containing a sequence for MDM4-A/S, many reads corresponding to MDM4-A expression were allocated to the MDM4-A/S isoform. It will be important to interpret previous data showing MDM4-A or MDM4-S expression as potentially inclusive of MDM4-A/S expression.

This study is limited primarily by sample size. In our previous work [23], we examined a larger number of FFPE melanoma specimens. These preserved samples have the advantage of being readily available from local dermatopathology clinics but the disadvantage of having highly fragmented nucleic acids; thus, they do not lend themselves to long-read sequencing. For that, we needed freshly frozen specimens. We approached the local branch of the Cooperative Human Tissue Network about this, and they were able to find only nine specimens of fresh frozen melanoma tissues. These are the nine that were utilized in the current study. Any larger study, like we hope to perform as a follow-up to this work, will need to prospectively collect specimens. Secondly, while we know that each specimen represents a stage IV (metastatic) tumor, the complete clinical data we have available are presented in Table 1. Several data points are regrettably absent, such as the Breslow index, but this is the nature of the anonymously donated tissues. Even the excision date is limited to the year to prevent attempts to reconnect specimens to specific patients. Again, a larger prospective study could, with appropriate institutional review board oversight, collect specimens with these data intact. A third limitation is that we do not present expression data on MDM2, TP53, or other genes in the p53 pathway, nor do we have mutation data on TP53 itself. A preliminary transcriptomic study was carried out with these specimens using nanopore sequencing. However, because of multiplexing resulting from budget limitations, the sequencing depth was insufficient to make conclusions about the expression of these genes. Furthermore, the error rate of nanopore sequencing prevented confident genotyping of TP53 from limited reads. While our previous work suggests that MDM2 is not frequently amplified in melanoma compared to MDM4, it will be of interest to pursue whether the p53 status and/or reliance on MDM2 expression correlates to the patterns of isoform expression of MDM4. 

Taken together, significant findings came from the current study, where MDM4-S was the most highly expressed transcript in nine malignant melanoma tumors, MDM4-209 was determined to be a relatively significant isoform, and a novel transcript was discovered through the use of long-read nanopore sequencing. The nature of MDM4 and its isoforms becomes more complex through these findings, stressing the need for more research on MDM4, the alterations in the p53 pathway as a result, and the functions of the MDM4-A/S transcript. The inhibition of MDM4 remains a “key therapeutic target in cutaneous melanoma”, potentially through alterations in the splicing landscape and resulting functional changes. Placing isoform expression into the larger context of mutation and transcriptional alterations in the p53 pathway remains an important future direction. 

## 4. Materials and Methods

### 4.1. Melanoma Sample Collection and RNA Extraction

Immersion frozen tissue samples of human tumors from metastatic melanomas were received from the Cooperative Human Tissue Network, which is funded by the National Cancer Institute. Other investigators may have received specimens from the same subjects. The metadata obtained for all 9 samples is showcased in Supplementary Table S1 with weight, diagnoses, gender, age, ethnicity, and method of preservation.

RNA was extracted from each tissue sample using the E.Z.N.A Total RNA Kit 1 by Omega Biotek (Norcross, GA, USA). RNA was then quantified using a nanodrop spectrophotometer, and the quality was assessed by an A_260_/A_280_ ratio of approximately 2.0 on an Agilent 2100 Bioanalyzer, following RNA nano chip preparation, using the RNA 6000 Nano Kit Guide by Agilent Technologies (Santa Clara, CA, USA).

### 4.2. cDNA Synthesis

RNA was reverse transcribed from each sample using random hexamers or oligo d(T) primer using The SuperScript IV CellsDirect cDNA Synthesis Kit from Thermo Fisher Scientific (Waltham, MA, USA). Reverse transcription using random hexamers is less specific to enable unbiased binding to mRNA, rRNA, and degraded RNA, therefore, yielding a higher concentration of cDNA, and is used for the downstream RT-PCR [59]. However, oligo d(T) primer only anneals to the poly(A) tails of mRNA and is used for downstream qPCR and flow cell sequencing library preparation [59]. All reverse-transcribed RNA (RT) reactions were diluted 1:5 with nuclease-free water and stored at −20 °C.

### 4.3. RT-PCR

RT-PCR was performed for all samples and isoforms depicted in Figure 2, except for MDM4-209, using the primer pairs listed in Supplementary Table S2. RT reactions using random hexamers per sample were prepared in accordance with Promega (Madison, WI, USA) GoTaq Green Master Mix Protocol for a 25 μL reaction volume: 12.5 μL of GoTaq Green Master Mix, 1 μL of upstream primer (10 μM), 1 μL of downstream primer (10 μM), 2 μL of DNA template (10 ng), and 8.5 μL of nuclease-free water. Optimized PCR was performed for 2 min at 95 °C (initial denaturation), 30 amplification cycles of 30 s at 95 °C, 30 s at 57 °C (annealing), 30 s at 73 °C, and a final extension of 5 min at 73 °C. The RT-PCR products underwent gel electrophoresis for product visualization. A positive (beta-actin) control and a negative (no DNA) control were loaded alongside the RT products.

### 4.4. RT-qPCR

qPCR was performed for all samples using the primer pairs listed in Supplementary Table S2 for expression of the MDM4 isoforms. The RT reactions using oligo d(T) primer per sample were prepared in accordance with the PowerUp SYBR Green Master Mix workflow (Applied Biosystems, Foster City, CA, USA) for a volume of 10 µL per well (Semi-Skirted 96-Well PCR Plate, USA Scientific, Ocala, FL, USA): 5 µL of PowerUp SYBR Green Master Mix, 1 μL of upstream primer (10 μM), 1 μL of downstream primer (10 μM), 2 μL of DNA template (10 ng), and 1 μL of nuclease-free water. The cycling conditions for all primer pairs, except MDM4-209, followed the cycling conditions for primer Tm < 60 °C. The cycling conditions for MDM4-209 followed the cycling conditions for primer Tm = or >60 °C. All qPCR was performed on a StepOnePlus Real-Time PCR System (Thermo Fisher Scientific, Waltham, MA, USA), and all PCR products were confirmed for the amplification of single products by the presence of single peaks on the dissociation/ melt curves. 

Isoform expression is relative to the beta-actin control and was quantified through a 2 ^(−ΔCT)^ analysis to obtain the relative expression. Then, the values were normalized to the overall isoform expression per sample. Overall, the relative expression of MDM4-209, MDM4-A, MDM4-FL, and MDM4-S isoforms were normalized to the total MDM4 expression per sample.

### 4.5. Flow Cell Sequencing Primer Design and Amplicon Purification

A primer pair was designed for use with flow cell sequencing to capture the maximum number of MDM4 isoforms possible: a forward primer within exon 1 (5′-GAGGCCCTAGGATCTGTGAC-3′) and reverse primer within exon 11 (5′-GGCTTCAAGAGATTCTGGCA-3′) of MDM4. The primers were designed using the MDM4-FL sequence from the Ensembl database to anneal to all isoforms depicted in Figure 2, alongside possibly undiscovered isoforms containing the primer annealing sequences. PCR reactions were performed for RT reactions using an oligo d(T) primer per sample, using Phusion High-Fidelity PCR Master Mix (Thermo Fisher Scientific, Waltham, MA, USA) for a 20 µL reaction volume: 10 μL of 2X Phusion Master Mix, 1 μL of forward primer (10 μM), 1 μL of reverse primer (10 μM), 2 μL of DNA template (10 ng), and 6 μL nuclease-free water. PCR was performed for 30 s at 98 °C, 30 amplification cycles (5 s at 98 °C, 20 s at 60 °C, and 30 s at 72 °C), and a final extension of 7 min at 72 °C. PCR products were then verified using gel electrophoresis for the indication of multiple bands per sample at the expected amplicon sizes for each isoform based on the primer design for the indication of multiple isoforms. 

Following PCR verification of the flow cell sequencing primers, all PCR amplicons were purified using the QIAquick PCR Purification Kit (Qiagen, Valencia, CA, USA) for the removal of impurities. 

### 4.6. cDNA Library Preparation for MinION Flow Cell Sequencing

The Native Barcoding Kit 24 V14 (SQK-NBD-114.24) from Oxford Nanopore Technologies (Oxford, England) was used to multiplex samples and prepare the purified PCR amplicons for nanopore sequencing. All amplicon cDNA was first “end-prepped”/repaired for 5′ phosphorylated and 3′ dA-tailed ends for the preparation of an adapter attachment using the NEBNext Ultra II End Repair/dA-Tailing Module (New England Biolabs Inc., Ipswich, MA, USA). Recommended starting amounts of end-prepped cDNA for the Native Barcode ligation step for short fragment libraries (amplicons or cDNA) was 100–200 fmol; however, due to a low yield of only 65 fmols per sample, this was carried over for Native Barcode ligation. End-prepped cDNA was determined to be of sufficient quantity by fluorometry using a Qubit hsDNA assay. Unique Native Barcodes (NB01–NB09) were ligated onto the end-prepped cDNA of each sample using NEB Blunt/TA Ligase Master Mix (New England Biolabs Inc., Ipswich, MA, USA). All reactions/samples were then pooled, and sequencing adapters were ligated onto the ends of the cDNA-pooled barcoded sample using the NEBNext Quick Ligation Module (New England Biolabs Inc., Ipswich, MA, USA). The final prepared library was diluted to 15 fmols (~10 ng) and stored at −20 °C overnight for flow cell loading.

### 4.7. MinION Flow Cell Priming and Library Loading

A MinION R10.4.1 flow cell (FLO-MIN114) was placed in the MinION sequencing device to perform a “Flow Cell Check” through the MinKNOW software interface version 24.02.6. The following sequencing parameters were input: R10.4.1 flow cell type, SQK-NBD-114.24 protocol type, no run-time limit, no minimum read length, Fast basecalling model, Barcode Trimming “on”, and default read filtering. Flow cell checks were successful, with greater than 800 active pores. 

The priming port was opened, and ~20 μL of buffer was drawn out without the introduction of air bubbles or the disruption of the array of pores. Flow cell priming mix was created using Flow Cell Flush, purified Bovine Serum Albumin (10 mg/ mL) (New England Biolabs Inc., Ipswich, MA, USA), and Flow Cell Tether. The priming mix was loaded through the Priming Port. A mixture of the previously prepared pooled barcoded cDNA library with Library Beads and Sequencing Buffer was loaded through the SpotON sample port. A light shield was immediately placed over the flow cell, and the MinKNOW device was closed to initiate sequencing.

### 4.8. Basecalling and Data Analysis through EPI2ME Labs Software

The basecalling results were displayed in real time through the Fast basecalling model of the MinKNOW software version 24.02.6 interface, with read files generated throughout the duration of the sequencing run. Raw reads were given as POD5 files, which can be re-basecalled through other basecalling models. Through the Fast basecalling model, data were written as FASTQ passed and FASTQ failed files through the default read calling filters and separated per barcode/sample. Oxford Nanopore Technologies sequencing devices follow the conventional Sanger FASTQ format to score reads on a Phred-scaled probability to designate “passed” and “failed” reads [60]. Only FASTQ-passed reads with the appropriate barcodes were used in the analyses. 

The EPI2ME Labs software (version 5.1.3) from ONT was utilized to de-multiplex reads and run alignment workflows using reference genomes, transcriptomes, and cDNA sequences obtained from the Ensembl genome browser database. EPI2ME Labs maintains and curates Nextflow (https://www.nextflow.io) (version 23.04.2) bioinformatics workflows through the integration of GitHub (https://github.com) code/pipelines with the use of Docker (https://www.docker.com) container technology. EPI2ME Labs provides predefined workflows and can pull bioinformatics pipelines from the GitHub repository (https://github.com/orgs/epi2me-labs/repositories), allowing both the creation and open public use of pipelines for data analysis. 

The wf-alignment workflow (version v0.6.0) was used for the creation of several alignment statistics. FASTQ-passed files from each sample and FASTA reference files were input using the default workflow filters. The workflow concatenated reference files (if the FASTA reference file was a path to a directory containing multiple references), aligned input reads to the combined reference using Minimap2, used Bamstats to create per-read and per-reference alignment stats, calculated the depth of coverage along the reference sequences using Mosdepth, and ultimately output an HTML report to illustrate the results (./wf-alignment-report.html), as a per-read and per-reference alignment statistics TSV file (./{{alias}}.readstats.tsv, ./{{alias}}.flagstat.tsv) and an alignment BAM file and alignment indexed file (./{{alias}}.sorted.aligned.bam, ./{{alias}}.sorted.aligned.bam.bai) for each sample with alignments of filtered input reads against the reference that was used in downstream data visualization and graph creation [61]. MDM4 specificity in our reads was verified by combined sample alignment to the human genomic reference (GRCh38.14) from the Ensembl genome browser. The GRCh38.14 FASTA reference was then trimmed to a chromosome 1 reference to further elucidate MDM4 specificity. An MDM4-FL cDNA FASTA reference file was obtained from Ensembl and trimmed to create a custom MDM4-FL reference based on the expected amplicon size and sequence from the primer pair used. All samples were then individually aligned to the custom MDM4-FL FASTA reference. An MDM4-A/S reference file was created by deleting exons 6 and 9 of the custom MDM4-FL reference file. All data for MDM4 isoform expression were created using a custom transcriptome FASTA reference file, with the sequences of each isoform represented in Supplementary Table S3, based on the expected amplicon sequences adapted from the complete cDNA sequences obtained from the Ensembl database. All mapping statistics (total reads, reads aligned, average read length, and percent alignment accuracy) were created using data obtained from the reports generated by the wf-alignment workflow.

### 4.9. Data Visualization through IGV 

The Java desktop application Integrative Genomics Viewer (IGV) (https://igv.org) (version 2.16.0) was used for the visualization of read alignment to reference files. IGV supports the visualization of large, diverse genomic and transcriptomic datasets, allowing real-time pan and zoom for all scales of resolution and giving insight into read sequence alignment and mutations or alteration from the reference (insertions/deletions, single nucleotide polymorphisms, copy number alterations, etc.) [52,53]. Since IGV displays data mapped to the genomic coordinates of a reference genome, the datasets loaded (BAM with BAM.bai files obtained from EPI2ME Labs wf-alignment workflow) corresponded to the same reference genomes/transcriptomes used in the upstream wf-alignment workflow [52]. For wf-alignment workflows where the FASTA reference file was a path to a directory containing multiple reference files, the wf-alignment combined reference output (./combined-refs.fasta) was used to load onto IGV. Once the FASTA reference files were loaded onto the IGV interface, the BAM and BAM.bai files were loaded as horizontal “tracks” appearing parallel to the reference. The National Center for Biotechnology Information (NCBI) Single Nucleotide Polymorphism (dbSNP) variation database was utilized for single nucleotide variant (SNV) characterization in the IGV data [53].

## Figures and Tables

**Table 1:** Alignment statistics of sample alignment to the amplicon-specific MDM4-FL cDNA.

Sample #	Total Reads	Mean Read Length	% Reads Aligned	% Alignment Accuracy
1	169,013	1189	85.1	87.3
2	190,585	1058	63.2	88.2
3	211,515	905	47	89.4
4	170,265	1033	65.3	89
5	185,109	1183	81.6	89.8
6	468,534	507	0 (55 reads)	89.2
7	328,836	655.8	11.8	89
8	190,170	845	33.4	91.2
9	133,644	1286	94.4	88.8
Average	227,519	962	60	89

The total reads output from the MinION sequencing run per sample is represented alongside the mean read lengths, the percentage of reads aligned, and the percentage alignment accuracy for each sample from the wf-alignment pipeline, using an MDM4-FL FASTA reference trimmed to match the expected amplicon.

## Data Availability

Sequencing data are archived at the NCBI Sequence Read Archive, BioProject ID: PRJNA1140143. BioSample accessions: SAMN42812609, SAMN42812610, SAMN42812611, SAMN42812612, SAMN42812613, SAMN42812614, SAMN42812615, SAMN42812616, and SAMN42812617.

## Supplementary Materials

The following supporting information can be downloaded at: https://www.mdpi.com/article/10.3390/ijms25179415/s1.
