# Supplementary material for: Long-Read MDM4 Sequencing Reveals Aberrant Isoform Landscape in Metastatic Melanomas
Source: Int J Mol Sci. 2024 Aug 30;25(17):9415. doi: 10.3390/ijms25179415 (PMC11395681; doi:10.3390/ijms25179415)

Supplementary Materials

Long-Read MDM4 Sequencing Reveals Aberrant Isoform Landscape in Metastatic Melanomas

Nehaal Patrick [1] and Michael Markey [1,*]

[1] Department of Biochemistry and Molecular Biology, Wright State University, Dayton, OH 45435, USA
* Correspondence: michael.markey@wright.edu

Supplementary Table S1. Tissue sample characterization and metadata. The 9 tissue samples obtained and used throughout the study are listed here with sample number, weight in grams, metastatic melanoma diagnosis, sex, age, ethnicity (all Caucasian) and mode of preservation.

Sample Number	Weight (g)	Diagnosis	Sex	Age	Ethnicity	Preservation
1	0.2	metastasis to lymph node	Male	67	White	LN2 immersed
2	0.8	metastasis to soft tissue	Female	77	White	LN2 immersed
3	0.32	metastasis to brain	Female	66	White	LN2 immersed
4	1.22	metastasis to lymph node	Female	46	White	LN2 vapor phase
5	1	metastasis to breast	Female	67	White	LN2 vapor phase
6	0.77	metastasis to lymph node	Female	69	White	LN2 immersed
7	0.49	Metastasis to lymph node	Female	56	White	LN2 immersed
8	0.25	metastasis to brain	Male	34	White	LN2 immersed
9	1.22	metastasis to ovary	Female	61	White	LN2 vapor phase

Supplementary Table S2. List of primer sequences and expected amplicon sizes. The forward and reverse primer sequences are listed in the 5′→3′ direction and overall expected amplicon sizes based on sequences obtained from the Ensembl genome browser database.

Primer Pair	Sequence 5′→3′	Amplicon Size
Beta-actin (forward)	TTCCTATGTGGGCGACGAG	229
B-actin (reverse)	GAAGGTCTCAAACATGATCTGG	
MDM4-FL (forward)	AGATGCTGCTCAGACTCTCG	347
MDM4-FL (reverse)	TGGCAGTACCCACATCCTGA	
MDM4-A (forward)	CACACTGCCTACCTCAGAGC	219
MDM4-A (reverse)	CCCACTTCAATCACCTGATTTGTC	
MDM4-S (forward)	CAGCAGGTGCGCAAGGTGAA	235
MDM4-S (reverse)	GCACTTTGCTGTAGTAGCAGTG	
MDM4-G (forward)	CCTGGACAAATCAATCAGGATCAC	127
MDM4-G (reverse)	AGGTAGGCAGTGTGGGGATA	
MDM4-Alt1 (forward)	CAGGTGCGCAAGGTGAAATG	240
MDM4-Alt1 (reverse)	TCCCACTTCAATCACCTGTAGT	
MDM4-Alt2 (forward)	ACTGTTAAAGAGGTGATTGAAGTGG	367
MDM4-Alt2 (reverse)	CCACTGAGTTGCAGGGATCA	
MDM4-211 (forward)	CTCCTGGACAAATCAATCAGGAAA	141
MDM4-211 (reverse)	CCACTGAGTTGCAGGGATCA	
MDM4-209 (forward)	GCCACTGCTACTACAGGATCA	131
MDM4-209 (reverse)	GCTCTGAGGTAGGCAGTGTG	

Supplementary Table S3. Expected MDM4 amplicon sequences used to create custom MDM4 transcriptome FASTA reference file. The Ensembl transcript identification names, common names and trimmed sequences used for each expected amplicon are listed. A hypothetical MDM4-A/S transcript sequence is also represented. Sequences were used to create custom MDM4 transcriptome FASTA files utilized in downstream alignment analyses. Sequences adapted from the Ensembl database.

Ensembl Transcript ID	Common Name	Sequence (trimmed)
ENST00000367182.8	MDM4-FL	GAGGCCCTAGGATCTGTGACTGCCACCCCTCCCCCCACCCGGGCTCGGCGGGGGAGCGACTCATGGAGCTGCCGTAAGTTTTACCAACAGACTGCAGTTTCTTCACTACCAAAATGACATCATTTTCCACCTCTGCTCAGTGTTCAACATCTGACAGTGCTTGCAGGATCTCTCCTGGACAAATCAATCAGGTACGACCAAAACTGCCGCTTTTGAAGATTTTGCATGCAGCAGGTGCGCAAGGTGAAATGTTCACTGTTAAAGAGGTCATGCACTATTTAGGTCAGTACATAATGGTGAAGCAACTTTATGATCAGCAGGAGCAGCATATGGTATATTGTGGTGGAGATCTTTTGGGAGAACTACTGGGACGTCAGAGCTTCTCCGTGAAAGACCCAAGCCCTCTCTATGATATGCTAAGAAAGAATCTTGTCACTTTAGCCACTGCTACTACAGATGCTGCTCAGACTCTCGCTCTCGCACAGGATCACAGTATGGATATTCCAAGTCAAGACCAACTGAAGCAAAGTGCAGAGGAAAGTTCCACTTCCAGAAAAAGAACTACAGAAGACGATATCCCCACACTGCCTACCTCAGAGCATAAATGCATACATTCTAGAGAAGATGAAGACTTAATTGAAAATTTAGCCCAAGATGAAACATCTAGGCTGGACCTTGGATTTGAGGAGTGGGATGTAGCTGGCCTGCCTTGGTGGTTTTTAGGAAACTTGAGAAGCAACTATACACCTAGAAGTAATGGCTCAACTGATTTACAGACAAATCAGGATGTGGGTACTGCCATTGTTTCAGATACTACAGATGACTTGTGGTTTTTGAATGAGTCAGTATCAGAGCAGTTAGGTGTTGGAATAAAAGTTGAAGCTGCTGATACTGAACAAACAAGTGAAGAAGTAGGGAAAGTAAGTGACAAAAAGGTGATTGAAGTGGGAAAAAATGATGACCTGGAGGACTCTAAGTCCTTAAGTGATGATACCGATGTAGAGGTTACCTCTGAGGATGAGTGGCAGTGTACTGAATGCAAGAAATTTAACTCTCCAAGCAAGAGGTACTGTTTTCGTTGTTGGGCCTTGAGGAAGGATTGGTATTCAGATTGTTCAAAGTTAACCCATTCTCTCTCCACGTCTGATATCACTGCCATACCTGAAAAGGAAAATGAAGGAAATGATGTCCCTGATTGTCGAAGAACCATTTCGGCTCCTGTCGTTAGACCTAAAGATGCGTATATAAAGAAAGAAAACTCCAAACTTTTTGATCCCTGCAACTCAGTGGAATTCTTGGATTTGGCTCACAGTTCTGAAAGCCAAGAGACCATCTCAAGCATGGGAGAACAGTTAGATAACCTTTCTGAACAGAGAACAGATACAGAAAACATGGAGGATTGCCAGAATCTCTTGAAGCC
ENST00000391947.6	MDM4-S	GAGGCCCTAGGATCTGTGACTGCCACCCCTCCCCCCACCCGGGCTCGGCGGGGGAGCGACTCATGGAGCTGCCGTAAGTTTTACCAACAGACTGCAGTTTCTTCACTACCAAAATGACATCATTTTCCACCTCTGCTCAGTGTTCAACATCTGACAGTGCTTGCAGGATCTCTCCTGGACAAATCAATCAGGTACGACCAAAACTGCCGCTTTTGAAGATTTTGCATGCAGCAGGTGCGCAAGGTGAAATGTTCACTGTTAAAGAGGTCATGCACTATTTAGGTCAGTACATAATGGTGAAGCAACTTTATGATCAGCAGGAGCAGCATATGGTATATTGTGGTGGAGATCTTTTGGGAGAACTACTGGGACGTCAGAGCTTCTCCGTGAAAGACCCAAGCCCTCTCTATGATATGCTAAGAAAGAATCTTGTCACTTTAGCCACTGCTACTACAGCAAAGTGCAGAGGAAAGTTCCACTTCCAGAAAAAGAACTACAGAAGACGATATCCCCACACTGCCTACCTCAGAGCATAAATGCATACATTCTAGAGAAGATGAAGACTTAATTGAAAATTTAGCCCAAGATGAAACATCTAGGCTGGACCTTGGATTTGAGGAGTGGGATGTAGCTGGCCTGCCTTGGTGGTTTTTAGGAAACTTGAGAAGCAACTATACACCTAGAAGTAATGGCTCAACTGATTTACAGACAAATCAGGATGTGGGTACTGCCATTGTTTCAGATACTACAGATGACTTGTGGTTTTTGAATGAGTCAGTATCAGAGCAGTTAGGTGTTGGAATAAAAGTTGAAGCTGCTGATACTGAACAAACAAGTGAAGAAGTAGGGAAAGTAAGTGACAAAAAGGTGATTGAAGTGGGAAAAAATGATGACCTGGAGGACTCTAAGTCCTTAAGTGATGATACCGATGTAGAGGTTACCTCTGAGGATGAGTGGCAGTGTACTGAATGCAAGAAATTTAACTCTCCAAGCAAGAGGTACTGTTTTCGTTGTTGGGCCTTGAGGAAGGATTGGTATTCAGATTGTTCAAAGTTAACCCATTCTCTCTCCACGTCTGATATCACTGCCATACCTGAAAAGGAAAATGAAGGAAATGATGTCCCTGATTGTCGAAGAACCATTTCGGCTCCTGTCGTTAGACCTAAAGATGCGTATATAAAGAAAGAAAACTCCAAACTTTTTGATCCCTGCAACTCAGTGGAATTCTTGGATTTGGCTCACAGTTCTGAAAGCCAAGAGACCATCTCAAGCATGGGAGAACAGTTAGATAACCTTTCTGAACAGAGAACAGATACAGAAAACATGGAGGATTGCCAGAATCTCTTGAAGCC
ENST00000454264.6	MDM4-A	GAGGCCCTAGGATCTGTGACTGCCACCCCTCCCCCCACCCGGGCTCGGCGGGGGAGCGACTCATGGAGCTGCCGTAAGTTTTACCAACAGACTGCAGTTTCTTCACTACCAAAATGACATCATTTTCCACCTCTGCTCAGTGTTCAACATCTGACAGTGCTTGCAGGATCTCTCCTGGACAAATCAATCAGGTACGACCAAAACTGCCGCTTTTGAAGATTTTGCATGCAGCAGGTGCGCAAGGTGAAATGTTCACTGTTAAAGAGGTCATGCACTATTTAGGTCAGTACATAATGGTGAAGCAACTTTATGATCAGCAGGAGCAGCATATGGTATATTGTGGTGGAGATCTTTTGGGAGAACTACTGGGACGTCAGAGCTTCTCCGTGAAAGACCCAAGCCCTCTCTATGATATGCTAAGAAAGAATCTTGTCACTTTAGCCACTGCTACTACAGATGCTGCTCAGACTCTCGCTCTCGCACAGGATCACAGTATATATTCCAAGTCAAGACCAACTGAAGCAAAGTGCAGAGGAAAGTTCCACTTCCAGAAAAAGAACTACAGAAGACGATATCCCCACACTGCCTACCTCAGAGCATAAATGCATACATTCTAGAGAAGATGAAGACTTAATTGAAAATTTAGCCCAAGATGAAACATCTAGGCTGGACCTTGGATTTGAGGAGTGGGATGTAGCTGGCCTGCCTTGGTGGTTTTTAGGAAACTTGAGAAGCAACTATACACCTAGAAGTAATGGCTCAACTGATTTACAGACAAATCAGGTGATTGAAGTGGGAAAAAATGATGACCTGGAGGACTCTAAGTCCTTAAGTGATGATACCGATGTAGAGGTTACCTCTGAGGATGAGTGGCAGTGTACTGAATGCAAGAAATTTAACTCTCCAAGCAAGAGGTACTGTTTTCGTTGTTGGGCCTTGAGGAAGGATTGGTATTCAGATTGTTCAAAGTTAACCCATTCTCTCTCCACGTCTGATATCACTGCCATACCTGAAAAGGAAAATGAAGGAAATGATGTCCCTGATTGTCGAAGAACCATTTCGGCTCCTGTCGTTAGACCTAAAGATGCGTATATAAAGAAAGAAAACTCCAAACTTTTTGATCCCTGCAACTCAGTGGAATTCTTGGATTTGGCTCACAGTTCTGAAAGCCAAGAGACCATCTCAAGCATGGGAGAACAGTTAGATAACCTTTCTGAACAGAGAACAGATACAGAAAACATGGAGGATTGCCAGAATCTCTTGAAGCC
ENST00000614459.4	MDM4-G	GAGGCCCTAGGATCTGTGACTGCCACCCCTCCCCCCACCCGGGCTCGGCGGGGGAGCGACTCATGGAGCTGCCGTAAGTTTTACCAACAGACTGCAGTTTCTTCACTACCAAAATGACATCATTTTCCACCTCTGCTCAGTGTTCAACATCTGACAGTGCTTGCAGGATCTCTCCTGGACAAATCAATCAGGATCACAGTATGGATATTCCAAGTCAAGACCAACTGAAGCAAAGTGCAGAGGAAAGTTCCACTTCCAGAAAAAGAACTACAGAAGACGATATCCCCACACTGCCTACCTCAGAGCATAAATGCATACATTCTAGAGAAGATGAAGACTTAATTGAAAATTTAGCCCAAGATGAAACATCTAGGCTGGACCTTGGATTTGAGGAGTGGGATGTAGCTGGCCTGCCTTGGTGGTTTTTAGGAAACTTGAGAAGCAACTATACACCTAGAAGTAATGGCTCAACTGATTTACAGACAAATCAGGATGTGGGTACTGCCATTGTTTCAGATACTACAGATGACTTGTGGTTTTTGAATGAGTCAGTATCAGAGCAGTTAGGTGTTGGAATAAAAGTTGAAGCTGCTGATACTGAACAAACAAGTGAAGAAGTAGGGAAAGTAAGTGACAAAAAGGTGATTGAAGTGGGAAAAAATGATGACCTGGAGGACTCTAAGTCCTTAAGTGATGATACCGATGTAGAGGTTACCTCTGAGGATGAGTGGCAGTGTACTGAATGCAAGAAATTTAACTCTCCAAGCAAGAGGTACTGTTTTCGTTGTTGGGCCTTGAGGAAGGATTGGTATTCAGATTGTTCAAAGTTAACCCATTCTCTCTCCACGTCTGATATCACTGCCATACCTGAAAAGGAAAATGAAGGAAATGATGTCCCTGATTGTCGAAGAACCATTTCGGCTCCTGTCGTTAGACCTAAAGATGCGTATATAAAGAAAGAAAACTCCAAACTTTTTGATCCCTGCAACTCAGTGGAATTCTTGGATTTGGCTCACAGTTCTGAAAGCCAAGAGACCATCTCAAGCATGGGAGAACAGTTAGATAACCTTTCTGAACAGAGAACAGATACAGAAAACATGGAGGATTGCCAGAATCTCTTGAAGCC
ENST00000616250.4	MDM4-Alt1	GAGGCCCTAGGATCTGTGACTGCCACCCCTCCCCCCACCCGGGCTCGGCGGGGGAGCGACTCATGGAGCTGCCGTAAGTTTTACCAACAGACTGCAGTTTCTTCACTACCAAAATGACATCATTTTCCACCTCTGCTCAGTGTTCAACATCTGACAGTGCTTGCAGGATCTCTCCTGGACAAATCAATCAGGTACGACCAAAACTGCCGCTTTTGAAGATTTTGCATGCAGCAGGTGCGCAAGGTGAAATGTTCACTGTTAAAGAGGTCATGCACTATTTAGGTCAGTACATAATGGTGAAGCAACTTTATGATCAGCAGGAGCAGCATATGGTATATTGTGGTGGAGATCTTTTGGGAGAACTACTGGGACGTCAGAGCTTCTCCGTGAAAGACCCAAGCCCTCTCTATGATATGCTAAGAAAGAATCTTGTCACTTTAGCCACTGCTACTACAGGTGATTGAAGTGGGAAAAAATGATGACCTGGAGGACTCTAAGTCCTTAAGTGATGATACCGATGTAGAGGTTACCTCTGAGGATGAGTGGCAGTGTACTGAATGCAAGAAATTTAACTCTCCAAGCAAGAGGTACTGTTTTCGTTGTTGGGCCTTGAGGAAGGATTGGTATTCAGATTGTTCAAAGTTAACCCATTCTCTCTCCACGTCTGATATCACTGCCATACCTGAAAAGGAAAATGAAGGAAATGATGTCCCTGATTGTCGAAGAACCATTTCGGCTCCTGTCGTTAGACCTAAAGATGCGTATATAAAGAAAGAAAACTCCAAACTTTTTGATCCCTGCAACTCAGTGGAATTCTTGGATTTGGCTCACAGTTCTGAAAGCCAAGAGACCATCTCAAGCATGGGAGAACAGTTAGATAACCTTTCTGAACAGAGAACAGATACAGAAAACATGGAGGATTGCCAGAATCTCTTGAAGCC
ENST00000612738.4	MDM4-Alt2	GAGGCCCTAGGATCTGTGACTGCCACCCCTCCCCCCACCCGGGCTCGGCGGGGGAGCGACTCATGGAGCTGCCGTAAGTTTTACCAACAGACTGCAGTTTCTTCACTACCAAAATGACATCATTTTCCACCTCTGCTCAGTGTTCAACATCTGACAGTGCTTGCAGGATCTCTCCTGGACAAATCAATCAGGTACGACCAAAACTGCCGCTTTTGAAGATTTTGCATGCAGCAGGTGCGCAAGGTGAAATGTTCACTGTTAAAGAGGTGATTGAAGTGGGAAAAAATGATGACCTGGAGGACTCTAAGTCCTTAAGTGATGATACCGATGTAGAGGTTACCTCTGAGGATGAGTGGCAGTGTACTGAATGCAAGAAATTTAACTCTCCAAGCAAGAGGTACTGTTTTCGTTGTTGGGCCTTGAGGAAGGATTGGTATTCAGATTGTTCAAAGTTAACCCATTCTCTCTCCACGTCTGATATCACTGCCATACCTGAAAAGGAAAATGAAGGAAATGATGTCCCTGATTGTCGAAGAACCATTTCGGCTCCTGTCGTTAGACCTAAAGATGCGTATATAAAGAAAGAAAACTCCAAACTTTTTGATCCCTGCAACTCAGTGGAATTCTTGGATTTGGCTCACAGTTCTGAAAGCCAAGAGACCATCTCAAGCATGGGAGAACAGTTAGATAACCTTTCTGAACAGAGAACAGATACAGAAAACATGGAGGATTGCCAGAATCTCTTGAAGCC
ENST00000367183.7	MDM4-211	GAGGCCCTAGGATCTGTGACTGCCACCCCTCCCCCCACCCGGGCTCGGCGGGGGAGCGACTCATGGAGCTGCCGTAAGTTTTACCAACAGACTGCAGTTTCTTCACTACCAAAATGACATCATTTTCCACCTCTGCTCAGTGTTCAACATCTGACAGTGCTTGCAGGATCTCTCCTGGACAAATCAATCAGGAAAATGAAGGAAATGATGTCCCTGATTGTCGAAGAACCATTTCGGCTCCTGTCGTTAGACCTAAAGATGCGTATATAAAGAAAGAAAACTCCAAACTTTTTGATCCCTGCAACTCAGTGGAATTCTTGGATTTGGCTCACAGTTCTGAAAGCCAAGAGACCATCTCAAGCATGGGAGAACAGTTAGATAACCTTTCTGAACAGAGAACAGATACAGAAAACATGGAGGATTGCCAGAATCTCTTGAAGCC
ENST00000463049.5	MDM4-209	GAGGCCCTAGGATCTGTGACTGCCACCCCTCCCCCCACCCGGGCTCGGCGGGGGAGCGACTCATGGAGCTGCCGTAAGTTTTACCAACAGACTGCAGTTTCTTCACTACCAAAATGACATCATTTTCCACCTCTGCTCAGTGTTCAACATCTGACAGTGCTTGCAGGATCTCTCCTGGACAAATCAATCAGGTACGACCAAAACTGCCGCTTTTGAAGATTTTGCATGCAGCAGGTGCGCAAGGTGAAATGTTCACTGTTAAAGAGGTCATGCACTATTTAGGTCAGTACATAATGGTGAAGCAACTTTATGATCAGCAGGAGCAGCATATGGTATATTGTGGTGGAGATCTTTTGGGAGAACTACTGGGACGTCAGAGCTTCTCCGTGAAAGACCCAAGCCCTCTCTATGATATGCTAAGAAAGAATCTTGTCACTTTAGCCACTGCTACTACAGGATCACAGTATGGATATTCCAAGTCAAGACCAACTGAAGCAAAGTGCAGAGGAAAGTTCCACTTCCAGAAAAAGAACTACAGAAGACGATATCCCCACACTGCCTACCTCAGAGCATAAATGCATACATTCTAGAGAAGATGAAGACTTAATTGAAAATTTAGCCCAAGATGAAACATCTAGGCTGGACCTTGGATTTGAGGAGTGGGATGTAGCTGGCCTGCCTTGGTGGTTTTTAGGAAACTTGAGAAGCAACTATACACCTAGAAGTAATGGCTCAACTGATTTACAGACAAATCAGGATGTGGGTACTGCCATTGTTTCAGATACTACAGATGACTTGTGGTTTTTGAATGAGTCAGTATCAGAGCAGTTAGGTGTTGGAATAAAAGTTGAAGCTGCTGATACTGAACAAACAAGTGAAGAAGTAGGGAAAGTAAGTGACAAAAAGGTGATTGAAGTGGGAAAAAATGATGACCTGGAGGACTCTAAGTCCTTAAGTGATGATACCGATGTAGAGGTTACCTCTGAGGATGAGTGGCAGTGTACTGAATGCAAGAAATTTAACTCTCCAAGCAAGAGGTACTGTTTTCGTTGTTGGGCCTTGAGGAAGGATTGGTATTCAGATTGTTCAAAGTTAACCCATTCTCTCTCCACGTCTGATATCACTGCCATACCTGAAAAGGAAAATGAAGGAAATGATGTCCCTGATTGTCGAAGAACCATTTCGGCTCCTGTCGTTAGACCTAAAGATGCGTATATAAAGAAAGAAAACTCCAAACTTTTTGATCCCTGCAACTCAGTGGAATTCTTGGATTTGGCTCACAGTTCTGAAAGCCAAGAGACCATCTCAAGCATGGGAGAACAGTTAGATAACCTTTCTGAACAGAGAACAGATACAGAAAACATGGAGGATTGCCAGAATCTCTTGAAGCC
N/A	MDM4-A/S	GAGGCCCTAGGATCTGTGACTGCCACCCCTCCCCCCACCCGGGCTCGGCGGGGGAGCGACTCATGGAGCTGCCGTAAGTTTTACCAACAGACTGCAGTTTCTTCACTACCAAAATGACATCATTTTCCACCTCTGCTCAGTGTTCAACATCTGACAGTGCTTGCAGGATCTCTCCTGGACAAATCAATCAGGTACGACCAAAACTGCCGCTTTTGAAGATTTTGCATGCAGCAGGTGCGCAAGGTGAAATGTTCACTGTTAAAGAGGTCATGCACTATTTAGGTCAGTACATAATGGTGAAGCAACTTTATGATCAGCAGGAGCAGCATATGGTATATTGTGGTGGAGATCTTTTGGGAGAACTACTGGGACGTCAGAGCTTCTCCGTGAAAGACCCAAGCCCTCTCTATGATATGCTAAGAAAGAATCTTGTCACTTTAGCCACTGCTACTACAGCAAAGTGCAGAGGAAAGTTCCACTTCCAGAAAAAGAACTACAGAAGACGATATCCCCACACTGCCTACCTCAGAGCATAAATGCATACATTCTAGAGAAGATGAAGACTTAATTGAAAATTTAGCCCAAGATGAAACATCTAGGCTGGACCTTGGATTTGAGGAGTGGGATGTAGCTGGCCTGCCTTGGTGGTTTTTAGGAAACTTGAGAAGCAACTATACACCTAGAAGTAATGGCTCAACTGATTTACAGACAAATCAGTGATTGAAGTGGGAAAAAATGATGACCTGGAGGACTCTAAGTCCTTAAGTGATGATACCGATGTAGAGGTTACCTCTGAGGATGAGTGGCAGTGTACTGAATGCAAGAAATTTAACTCTCCAAGCAAGAGGTACTGTTTTCGTTGTTGGGCCTTGAGGAAGGATTGGTATTCAGATTGTTCAAAGTTAACCCATTCTCTCTCCACGTCTGATATCACTGCCATACCTGAAAAGGAAAATGAAGGAAATGATGTCCCTGATTGTCGAAGAACCATTTCGGCTCCTGTCGTTAGACCTAAAGATGCGTATATAAAGAAAGAAAACTCCAAACTTTTTGATCCCTGCAACTCAGTGGAATTCTTGGATTTGGCTCACAGTTCTGAAAGCCAAGAGACCATCTCAAGCATGGGAGAACAGTTAGATAACCTTTCTGAACAGAGAACAGATACAGAAAACATGGAGGATTGCCAGAATCTCTTGAAGCC

Supplementary Figure S1. Research methodology. RNA was extracted from frozen tissue specimens. Targeted PCR was used to generate cDNA from multiple MDM4 isoforms, including potentially unknown isoforms. PCR success was verified by gel electrophoresis. These cDNA were then used for ONT library preparation and sequenced on a MinION instrument.

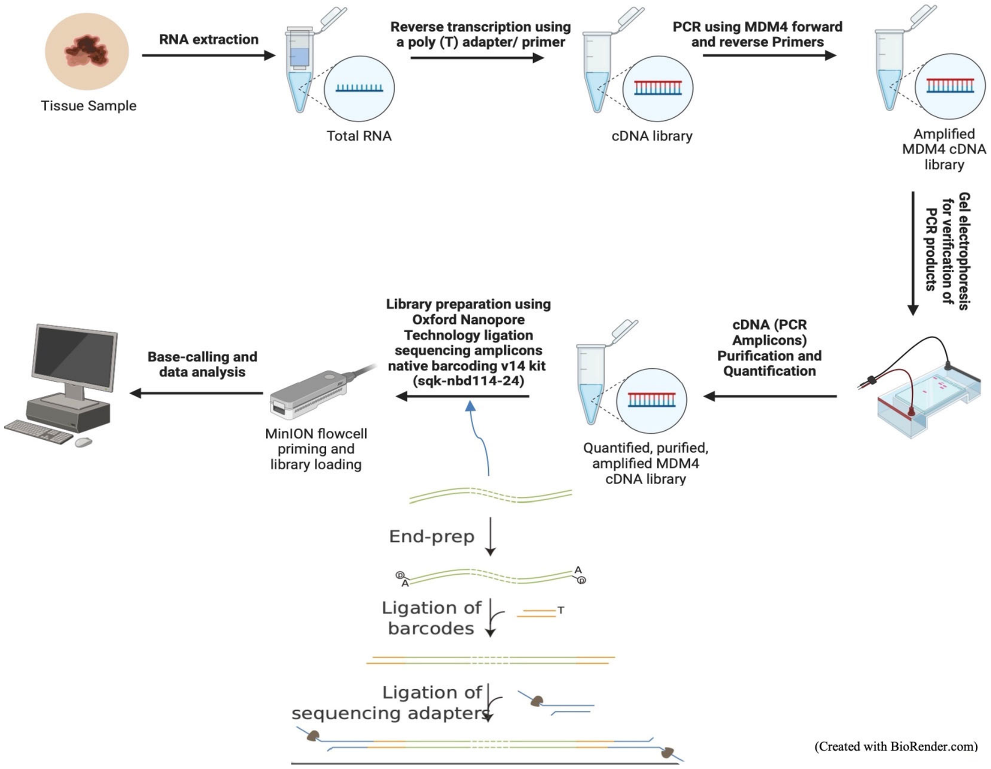

Supplement: Supplementary file 1 [file ijms-25-09415-s001.zip › ijms-3152024-supplementary.pdf]
